# An exploratory study on the association of multiple metals in serum with preeclampsia

**DOI:** 10.3389/fpubh.2024.1336188

**Published:** 2024-03-05

**Authors:** Jie He, Yudong Pu, Yue Du, Haixia Liu, Xiaoxue Wang, Shuzhen He, Shiwei Ai, Yuhui Dang

**Affiliations:** ^1^Institute of Maternal, Child and Adolescent Health, School of Public Health, Lanzhou University, Lanzhou, China; ^2^Songshan Lake Central Hospital of Dongguan City, Dongguan, China

**Keywords:** preeclampsia, metal, copper, logistic regression model, principal component analysis, Bayesian kernel machine regression

## Abstract

**Background:**

Individual metal levels are potential risk factors for the development of preeclampsia (PE). However, understanding of relationship between multiple metals and PE remains elusive.

**Purpose:**

The purpose of this study was to explore whether eight metals [zinc (Zn), manganese (Mn), copper (Cu), nickel (Ni), lead (Pb), arsenic (As), cadmium (Cd), and mercury (Hg)] in serum had a certain relationship with PE.

**Methods:**

A study was conducted in Dongguan, China. The concentrations of metals in maternal serum were assessed using inductively coupled plasma mass spectrometry (ICP-MS). Data on various factors were collected through a face-to-face interview and hospital electronic medical records. The unconditional logistic regression model, principal component analysis (PCA) and Bayesian Kernel Machine Regression (BKMR) were applied in our study.

**Results:**

The logistic regression model revealed that the elevated levels of Cu, Pb, and Hg were associated with an increased risk of PE. According to PCA, principal component 1 (PC1) was predominated by Hg, Pb, Mn, Ni, Cu, and As, and PC1 was associated with an increased risk of PE, while PC2 was predominated by Cd and Zn. The results of BKMR indicated a significant positive cumulative effect of serum metals on PE risk, with Ni and Cu exhibiting a significant positive effect. Moreover, BKMR results also revealed the nonlinear effects of Ni and Cd.

**Conclusion:**

The investigation suggests a potential positive cumulative impact of serum metals on the occurrence of PE, with a particular emphasis on Cu as a potential risk factor for the onset and exacerbation of PE. These findings offer valuable insights for guiding future studies on this concern.

## 1 Introduction

Preeclampsia (PE) is a pregnancy-specific complication with significant morbidity and mortality (1), and it stands out as one of the primary factors associated with maternal and perinatal death (2). Affecting 5–7% of all pregnant women, PE causes over 70,000 maternal deaths and 500,000 fetal deaths worldwide annually (1). Women with PE are usually at higher risk of placental abruption and intrauterine fetal death, as well as at higher risk of liver, kidney, brain, lungs and other organ diseases, which may further develop into eclampsia, cardiovascular and cerebrovascular diseases (3, 4). Furthermore, PE may also associated with adverse neonatal outcomes, including respiratory distress syndrome, retinopathy of prematurity, necrotizing enterocolitis, neurodevelopmental delay, and fetal or neonatal death (5). Characterized by abnormal vascular remodeling in the spiral arteries starting in the first trimester of pregnancy, PE results in placental hypoperfusion and release of various deleterious factors, which may trigger systemic endothelial response (6, 7). Despite this, the etiology of PE remains incompletely defined (8). Currently, there is no effective method to prevent or treat PE, and the primary recourse is abortion or delivery (9). In light of the above, more research is needed to unravel the etiology of PE to provide a foundation for prevention and novel treatment strategies. In view of industrial development, women are increasingly exposed to environmental toxicants, including a variety of metals, recognized as a significant risk factor for adverse pregnancy outcomes, such as spontaneous preterm birth and preeclampsia (10, 11).

In order to mitigate the impact of metal pollution on human health, numerous studies have been conducted to further investigate metals, including the exploration of novel adsorbents for the removal of metal ions from contaminated water (12), and the therapeutic potential of metal dithiocarbamate complexes in certain diseases (13). Expanding new ideas for mitigating metal health hazards and the application of metals in health, however, it is crucial not to overlook the hazards posed by metal exposure. Elucidating the health effects resulting from such exposure remains a pivotal area of research. The term “heavy metal” is a general classification for metals and metalloids with relatively high density and are considered toxic to living organisms and the environment at certain concentrations (14, 15). Lead (Pb), arsenic (As), cadmium (Cd), and mercury (Hg) are some examples of toxic heavy metals. Pb exposures has been shown to affect reproductive, hepatic, endocrine, immune and gastrointestinal systems (16). As is a recognized neurotoxin and is classified as human carcinogen, causing reproductive and developmental problems, as well as damages to the skin, digestive and respiratory systems (17). Cd can accumulate in kidney, liver, bones and other organs, causing damage to the target organs (18). Hg may cause damages to the brain, gut lining, kidneys, lungs and other vital organs, while low-grade chronic exposures to Hg may also induce subtler symptoms and clinical findings (19). Zinc (Zn), manganese (Mn), copper (Cu), and nickel (Ni) are essential trace metals vital for many physiological functions. Severe Zn deficiency may result in pustular dermatitis, alopecia, diarrhea and other symptoms (20), whereas, Mn serve as co-factor for many enzymes such as arginase, glutamine synthetase, manganese superoxide dismutase enzymes, etc. (21). Cu is also required for the catalytic function of several crucial cellular enzymes (22). However, higher levels of Zn can lead to Cu deficiency or anemia (20), while Mn is toxic to humans when exposed to certain concentrations (21). Excessive Cu exposures could also harm cells as it potentially catalyze the generation of toxic reactive oxygen species (ROS) (22). Ni is widely present in nature (23), and Ni exposures can cause a variety of adverse effects to human health, such as allergy, cardiovascular and kidney diseases, lung fibrosis, lung and nasal cancer (24, 25).

Previous studies have indicated significant relationships between the Cu (26) and Pb (27) levels in the maternal circulation and PE. Higher Mn (28, 29) and Zn (30, 31) levels were associated with lower risk of PE, whereas, elevated Cd (32), As (33) and Hg (34) levels, on the other hand, could potentially increase the risk of PE. Most of previous studies have focused to explore relationships between individual metal exposure and PE, however, the aforementioned toxic heavy metals concurrently present in environment and pregnant women are generally exposed to a variety of these metals simultaneously. Therefore, it is essential to explore the relationship between multiple metals and PE. Few studies have reported the association of multiple metals with PE (35, 36), however the results vary significantly and more research is needed to elucidate exact nature of this relationship.

Current study was conducted in Dongguan city, situated southeast of Guangdong Province, China. It is one of the world’s largest electronics manufacturing centers, with severe water, air and soil pollution. Excessive industrial emissions in the past few decades have resulted in elevated levels of heavy metals in soil (37, 38). Several studies have reported low to high level pollution of Cd, Cu, Hg, Ni, Pb, and Zn, and local children are facing a slight threat from As and chromium (Cr) mainly through oral ingestion of soil particles (39). Therefore, this research selected pregnant women who lived in Dongguan city for more than 1 year, and determined the concentrations of eight metals [zinc (Zn), manganese (Mn), copper (Cu), nickel (Ni), lead (Pb), arsenic (As), cadmium (Cd), and mercury (Hg)] in their peripheral venous blood to explore whether these metals were related to the occurrence of PE.

Previous studies have predominantly investigated the correlation between individual metals and PE (32–34, 40–42). Furthermore, investigations exploring multiple metals have infrequently delved into their interactions (36, 43), and the results have been inconsistent. In our study, pregnant women were recruited from a typical metal-contaminated area to scrutinize the relationship between multiple metal exposures and PE. We employed more appropriate and diverse methods to assess the prominence of specific metals in relation to PE and to explore potential interactions among multiple metals, thereby providing additional evidence on the mechanisms underlying the impact of metals on PE, offering novel insights for the prevention and treatment of PE.

## 2 Materials and methods

### 2.1 Study population

The study population was sourced from pregnant women attending Songshan Lake Central Hospital of Dongguan City in Guangdong Province, China, during the period from January 1, 2017 to December 31, 2017, specifically those who were at the hospital for delivery. Pregnant women aged ≥18 years, without diagnosed mental illness, and living in Dongguan city for more than 1 year were eligible to participate in this study. Women were diagnosed with PE based on the following criteria (2013): new-onset hypertension (SBP ≥ 140 mmHg, or DSP ≥ 90 mmHg) on two occasions at least 4 h apart and proteinuria (≥300 mg/24 h) after 20 weeks of gestation. PE cases were categorized into mild PE or severe PE, women appeared one of the following characteristics were included in the severe PE group: blood pressure (SBP ≥ 160 mmHg, or DBP ≥ 110 mmHg), proteinuria (5,000 mg/24 h), thrombocytopenia, liver dysfunction, renal insufficiency, pulmonary edema, visual impairment, new type headache and no response to drugs. A total of 271 pregnant women were willing to participate in our study, of whom 97 were diagnosed with mild PE, 64 with severe PE, and 110 were normotensive healthy pregnant women. Pregnancies resulting from *in vitro* fertilization, or women with preexistent hypertension, diabetes mellitus, kidney disease, cancer, severe anemia or other endocrine disorders were excluded. Women who did not donate blood samples or had missing data on crucial parameters were also excluded. Finally, our research comprised 28 cases of mild PE, 28 cases of severe PE and 28 normotensive healthy pregnant women. Written informed consent was obtained from all eligible participants. This study was approved by the Ethics Committee of the Songshan Lake Central Hospital of Dongguan City and the Ethics Committee of the School of Public Health of Lanzhou University.

### 2.2 Sample collection

The demographic characteristics of participants were obtained through face-to-face interview covering details such as age, occupational status, education, marital status and blood type. Information on physician diagnoses, gravidity, parity, gestational age and other diseases history were extracted from hospital electronic medical records at the hospital. Fasting peripheral venous blood samples were collected within 24 h before delivery, and then serum extracted from these samples were collected and stored at −80°C for further analysis. Blood samples were digested by the microwave digestion system (PreeKem, TOPEX+, China). Briefly, 0.5 mL blood sample and 3 mL HNO_3_ were added into the digestion tank, with the following temperature–time regimen: 100°C-3 min, 130°C-3 min, 160°C-3 min, and 190°C-20 min. Following digestion, concentrations of metals in blood samples were determined by inductively coupled plasma-mass spectrometry (ICP-MS) (44). Quality control measures were taken into consideration by using blanks, three parallel samples and a standard reference material [GBW (E) 080067]. The relative standard deviations of the parallel samples were <10%, and the recovery of standard reference material was 94%.

### 2.3 Calculation

Continuous variables were presented as mean ($\bar{x}$) ± standard deviation (*SD*), while categorical variables were expressed by number (*N*) and percentage (%). To compare categorical variables, Pearson chi-square test and Fisher exact test were used. The Kruskal–Wallis *H* test was utilized for the comparison of non-normally distributed continuous variables. The Spearman’s rank correlation was used to probe the associations of metal concentrations.

Unconditional logistic regression models were employed to calculate the odds ratios (ORs) and 95% confidence intervals (95% CIs). A univariate model explored the associations between single metals and PE, while a multivariate model examined the associations between multiple metals and PE. Each model was adjusted for potential confounders, specifically maternal education and gestational age, given the comparison of these factors were was statistically significant (*p* < 0.05) between different groups. Associations between PE and an overall measure of exposure to eight metals was derived by summing the quartile category score (1–4, with 1 representing the lowest quartile) for each metal to create an overall score with values ranging from 8 to 32 were estimated (45).

Principal component analysis (PCA) was employed to investigate the collective effects of multiple metals (46). PCA was intended to select clusters and characterized similar metals into new composite variables, called principal components (PCs). Loading factors that indicate the importance of the metals in a specific PC were estimated through varimax rotation, and the eigenvalues and the scree test determined the suitable number of PCs. The relationship between these PCs and PE was investigated using multivariate logistic regression.

Bayesian kernel machine regression (BKMR) was utilized to determine potential nonlinear effects of metals on PE and interactions among metals. Concentrations of metal were log-transformed to address skewed data after scaling. In the study, the BKMR model follows: Y_i_ = h (Zn, Mn, Ni, Cu, Pb, As, Cd, Hg) + β^T^Z_i_ + e_i_, whereas the function h() is a dose–response function, and Z1, …, Zp are p potential confounders. The cumulative and single effects of the eight metals were plotted by comparing the estimated value of the exposure-response function when all of the other metals were at a particular quantile. In addition, a dose–response relationship of each metal with PE was plotted while fixing the rest of metals at their 50th percentile to show the nonlinear relationship. The bivariate exposure-response function for two metals was also visualized, with all of the other metals fixed at their median value, indicating potential interaction between the two metals. Furthermore, a hierarchical variable selection method was applied, and metals were divided into two groups due to the results of PCA.

The statistical analyses were performed using SPSS (version 26) and R studio software (R version 4.3.0). A two-sided *p-*value of less than 0.05 was considered statistically significant, indicating a significant difference between variables.

## 3 Results

### 3.1 Participant characteristics

The results indicated statistically significant differences in maternal education and gestational age among three groups (*p* < 0.05). There was significant difference between the control group and the mild PE group in the middle school stratification (*p* < 0.05), with women in the mild PE group having lower education level than controls. In the high school or above stratification, significant differences were observed between the control group and both the mild and severe PE groups (*p* < 0.05), with women in the control group being more educated than those with mild or severe PE. Overall, women who experienced PE were found to be less educated as compared to those without PE. Gestational age showed statistically significant differences between the control or mild PE group and the severe PE group (*p* < 0.01), with women in severe PE group having shorter gestational ages. No statistically significant differences were observed among the three groups in maternal age, occupational status, marital status, blood type, gravidity and parity. Detailed demographic characteristics of the participants are shown in Table 1.

**Table 1:** Demographic characteristics of participants.

Characteristics	Controls (*N* = 28)	Mild PE (*N* = 28)	Severe PE (*N* = 28)	*P*-value
Maternal age, years ($\bar{x}$±*SD*)	30.54 ± 5.11	29.07 ± 5.76	30.46 ± 7.00	0.342^3^
Occupational status, *n* (%)				
Unemployment	15 (53.60)	20 (71.40)	19 (67.90)	0.337^1^
Employment	13 (46.40)	8 (28.60)	9 (32.10)	
Marital status, *n* (%)				
Unmarried	1 (3.60)	3 (10.70)	4 (14.30)	0.520^2^
Married	27 (96.40)	25 (89.30)	24 (85.70)	
Maternal education, *n* (%)				
Primary school or below	2 (7.10)^a^	2 (7.10)^a^	5 (17.90)^a^	0.028^2^
Middle school	12 (42.90)a	21 (75.00)^b^	18 (64.30)^a,b^	
High school or above	14 (50.00)^a^	5 (17.90)^b^	5 (17.90)^b^	
Blood type, *n* (%)				
A	12 (42.90)	12 (42.90)	8 (28.60)	0.505^2^
B	4 (14.30)	4 (14.30)	6 (21.40)	
O	8 (28.60)	8 (28.60)	13 (46.40)	
AB	4 (14.30)	4 (14.30)	1 (3.60)	
Gravidity, *n* (%)				
1	7 (25.00)	11 (39.30)	12 (42.90)	0.445^1^
≥2	21 (75.00)	17 (60.70)	16 (57.10)	
Parity, *n* (%)				
Primiparous	11 (39.30)	17 (60.70)	17 (60.70)	0.233^1^
Multiparous	17 (60.70)	11 (39.30)	11 (39.30)	
Gestational age, weeks ($\bar{x}$±SD)	38.53 ± 1.37^a^	38.82 ± 1.61^a^	36.67 ± 2.62^b^	0.001^3^

The same letter indicates no statistical difference between different groups. On the contrary, different letters indicate that the comparisons between different groups were statistically significant (such as a, b, and c). $\bar{x},$ mean; SD, standard deviation. ^1^Pearson chi-square test. ^2^Fisher exact test. ^3^Kruskal–Wallis *H* test.

### 3.2 Concentrations of metal in serum of participants

**Figure 1:**
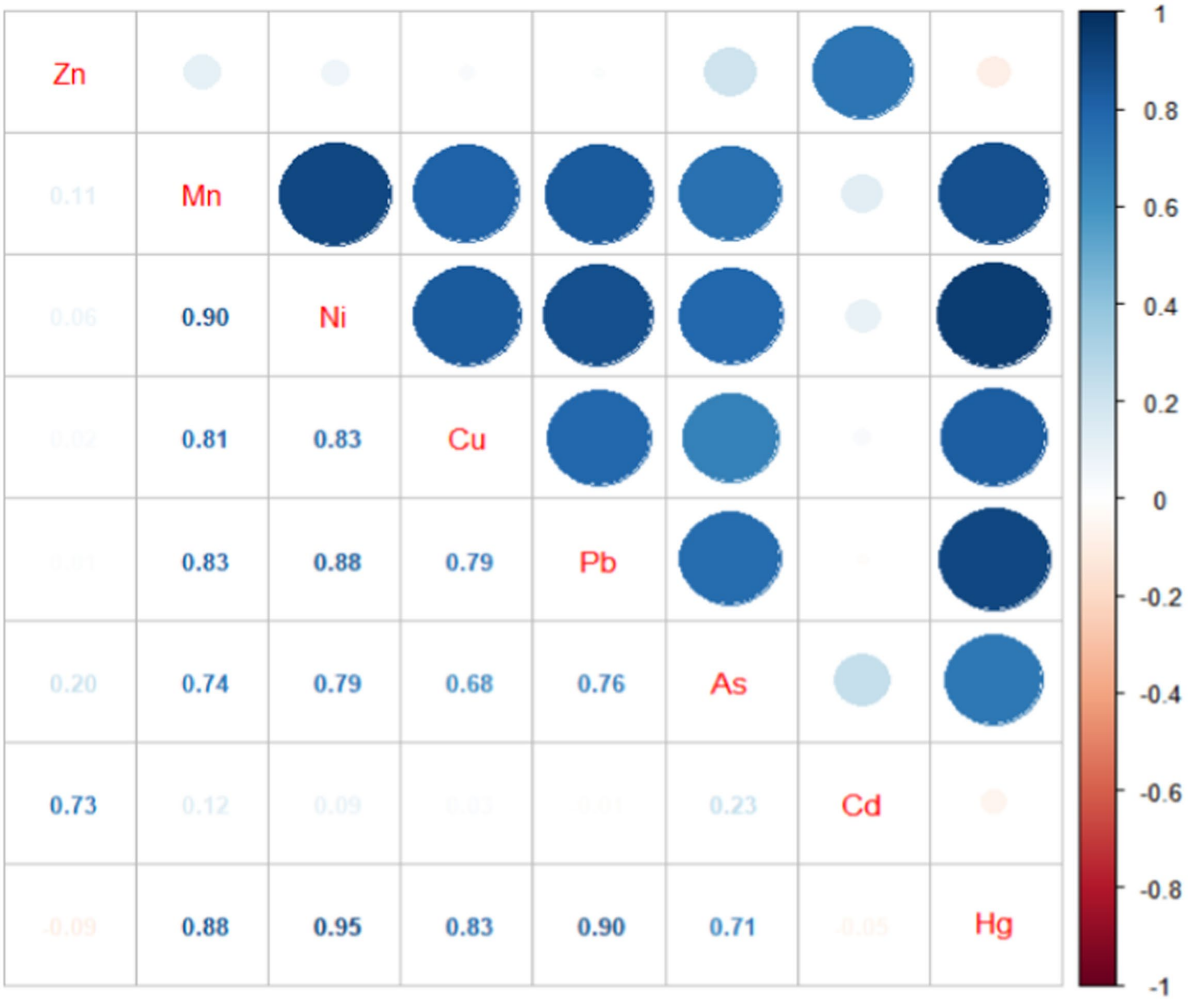
Correlation map of the serum metals.

Table 2 shows concentrations of metal measured in maternal serum. There were statistically significant differences in the concentrations of metals among the three groups (*p* < 0.01). Specifically concentrations of Mn, Ni, Cu, Pb, As, and Hg gradually increased from the control group to the severe PE group. However, concentrations of Zn and Cd in the severe PE group were lower than the those in control and the mild PE groups. Figure 1 illustrates the correlation analysis results between concentrations of metal in maternal serum. The Spearman correlation between blood concentrations of Hg, Mn, Ni, Cu, and Pb was highly significant with each other (*p* < 0.01). Additionally, blood concentrations of As correlated with Hg, Mn, Ni, Cu, Pb, and Cd (*p* < 0.05), while Zn correlated with Cd (*p* < 0.01).

**Table 2:** Concentrations of metal among three groups.

Metals (μg/L)	Controls	Mild PE	Severe PE	*P*-value
	Median (IQR)	Median (IQR)	Median (IQR)	
Zn	5794.85 (4747.16, 6940.79)^a^	7502.32 (6822.97, 9390.16)^a^	3956.40 (2808.63, 6051.72)^b^	0.000
Mn	4176.40 (3812.12, 4696.05)^a^	4980.18 (4565.15, 5163.88)^b^	8118.92 (5347.99, 9418.99)^c^	0.000
Ni	2893.60 (76.65, 3453.47)^a^	3897.13 (3667.93, 4038.01)^b^	6440.99 (4475.77, 7777.19)^c^	0.000
Cu	1363.93 (1283.23, 1509.60)^a^	1939.52 (1768.27, 2006.59)^b^	2840.72 (2536.13, 3131.70)^c^	0.000
Pb	122.38 (72.73, 152.61)^a^	162.44 (153.10, 182.31)^b^	344.45 (249.50, 452.75)^c^	0.000
As	10.21 (4.93, 12.28)^a^	12.53 (10.91, 14.52)^b^	18.42 (12.70, 24.13)^c^	0.000
Cd	5.97 (2.31, 8.57)^a^	10.11 (8.85, 12.38)^b^	2.29 (1.74, 4.13)^a^	0.000
Hg	5.05 (2.30, 6.15)^a^	6.58 (6.21, 6.95)^b^	13.20 (9.35, 16.07)^c^	0.000

The same letter indicates no statistical difference between different groups. On the contrary, different letters indicate that the comparisons between different groups were statistically significant (such as a, b, and c; IQR, interquartile range; *P-*value by Kruskal–Wallis *H* test.).

### 3.3 Unconditional logistic regression analyses

The ORs and 95% CIs for PE in relationship to concentrations of single metal after adjustment for potential confounders are presented in Table 3. The results indicated that elevated concentrations of Mn, Cu, Pb, and Hg were associated with the risk and severity of PE. Specifically, the increased concentration of Zn was associated with an increased risk of PE in the mild PE group (OR = 6.66, 95% CI: 1.82, 24.40 for the high vs. low group), while, elevated concentration of As was associated with an increased risk of PE in the severe PE group (OR = 15.79, 95% CI: 3.51, 70.98 for the high vs. low group). The elevated concentration of Cd was associated with an increased risk of PE in the mild PE group (OR = 24.16, 95% CI: 3.84, 151.82 for the high vs. low group), however, in the severe PE group, the elevated concentration of Cd was associated with lower risk of PE (OR = 0.08, 95% CI: 0.01, 0.47 for the high vs. low group). The overall score, derived from the cumulative effects of eight metals, was associated with the risk and severity of PE after adjustment (*p* < 0.01) (Table 3). The high overall score was associated with an increased risk of PE (OR = 11.00, 95% CI: 2.71, 44.66 and 19.99, 4.20, 95.21 for the high vs. low group, in the mild PE group and the severe PE group, respectively).

**Table 3:** Associations between single metals and PE.

Metals (μg/L)	Mild PE		Severe PE	
	OR (95%CI)	*P*-value	OR (95%CI)	*P*-value
Zn				
Low (<6111.84)	1.00	–	1.00	–
High (≥6111.84)	6.66(1.82,24.40)	0.004	0.53(0.15,1.89)	0.325
Mn				
Low (<4991.59)	1.00	–	1.00	–
High (≥4991.59)	5.03(1.23,20.61)	0.025	40.39(6.97,234.06)	0.000
Ni				
Low (<3804.87)	1.00	–	1.00	–
High (≥3804.87)	9.86E+08(2.10E+08,4.63E+09)	0.000	3.53E+09(3.53E+09,3.53E+09)	–
Cu				
Low (<1926.55)	1.00	–	1.00	–
High (≥1926.55)	34.96(3.94,310.61)	0.001	227.64(19.06,2718.40)	0.000
Pb				
Low (<165.15)	1.00	–	1.00	–
High (≥165.15)	7.07(1.53,32.72)	0.012	334.13(24.27,4601.10)	0.000
As				
Low (<12.40)	1.00	–	1.00	–
High (≥12.40)	2.50(0.72,8.68)	0.148	15.79(3.51,70.98)	0.000
Cd				
Low (<6.55)	1.00	–	1.00	–
High (≥6.55)	24.16(3.84,151.82)	0.001	0.08(0.01,0.47)	0.005
Hg				
Low (<6.58)	1.00	–	1.00	–
High (≥6.58)	11.33(2.13,60.19)	0.004	278.87(24.38,3189.41)	0.000
Overall score				
Low (<21.00)	1.00	–	1.00	–
High (≥21.00)	11.00(2.71,44.66)	0.001	19.99(4.20,95.21)	0.000

Adjusted for maternal education and gestational age.

Table 4 presents the ORs and 95% CIs for PE in relationship to concentrations of multiple metals after adjustment for potential confounders. An increased risk of PE was associated with elevated concentrations of Cu (OR = 45.37, 95% CI: 3.11, 661.15 and 159.09, 4.53, 5590.15 for the high vs. low group, in the mild PE group and the severe PE group, respectively), and Pb (OR = 8.82, 95% CI: 1.08, 71.90 and 1052.89, 17.57, 63106.99 for the high vs. low group, in the mild PE group and the severe PE group, respectively). Additionally, the elevated concentration of Hg (OR = 172.07, 95% CI: 2.96, 10021.03 for the high vs. low) was associated with an increased risk of PE in the severe PE group.

**Table 4:** Associations between multiple metals and PE.

Metals (μg/L)	Mild PE		Severe PE	
	OR (95%CI)	*P-*value	OR (95%CI)	*P*-value
Mn				
Low (<4991.59)	1.00	–	1.00	–
High (≥4991.59)	0.49(0.06,4.20)	0.515	1.40(0.09,21.12)	0.809
Cu				
Low (<1926.55)	1.00	–	1.00	–
High (≥1926.55)	45.37(3.11,661.15)	0.005	159.09(4.53,5590.15)	0.005
Pb				
Low (<165.15)	1.00	–	1.00	–
High (≥165.15)	8.82(1.08,71.90)	0.042	1052.89(17.57,63106.99)	0.001
Hg				
Low (<6.58)	1.00	–	1.00	–
High (≥6.58)	8.43(0.87,82.08)	0.066	172.07(2.96,10021.03)	0.013

Adjusted for maternal education and gestational age.

### 3.4 Principal component analyses

As shown in Table 5, two PCs were identified through PCA. The first PC (PC1) was predominated by Hg, Pb, Mn, Ni, Cu, and As, while the second PC (PC2) was predominated by Cd and Zn. The ORs and 95% CIs for PE in relationship to PCs, both before and after adjustment for potential confounders, are shown in Table 6. After adjustment, PC1 was associated with an increased risk of PE (OR = 47.97, 95% CI: 4.64, 496.26 and 432.69, 28.17, 6645.03 for the high vs. low, in the mild and severe PE group, respectively), while PC2 was associated with an increased risk of PE (OR = 21.72, 95% CI: 3.61,130.75 for the high vs. low) in the mild PE group, and with lower risk of PE (OR = 0.05, 95% CI: 0.01, 0.46 for the high vs. low) in the severe PE group.

**Table 5:** Principal component analysis results of metals.

Metals	PC 1	PC 2
Hg	0.991	0.046
Pb	0.990	0.040
Mn	0.965	0.124
Ni	0.955	0.143
Cu	0.943	0.090
As	0.894	0.304
Cd	0.107	0.908
Zn	0.101	0.894
Characteristic root	5.515	1.764
Percentage of total variance	68.941	22.051
Cumulative contribution rate	68.941	90.992

Used the rotated component matrix. PC, principal component.

**Table 6:** Associations between PCs and PE.

Model	PCs	Mild PE		Severe PE	
		OR (95%CI)	*P*-value	OR (95%CI)	*P*-value
Model 1	PC 1				
	<−0.77	1.00	–	1.00	–
	≥−0.77	36.00(4.27,303.44)	0.001	225.00(21.94,2307.07)	0.000
	PC 2				
	<−0.08	1.00	–	1.00	–
	≥−0.08	15.00(2.97,75.70)	0.001	0.09(0.02,0.45)	0.003
Model 2	PC 1				
	<−0.77	1.00	–	1.00	–
	≥−0.77	47.97(4.64,496.26)	0.001	432.69(28.17,6645.03)	0.000
	PC 2				
	<−0.08	1.00	–	1.00	–
	≥−0.08	21.72(3.61,130.75)	0.001	0.05(0.01,0.46)	0.009

Model 1 was an unadjusted model. Model 2 was adjusted for maternal education and gestational age.

### 3.5 Bayesian kernel machine regression analyses

A BKMR model was employed to assess the effect of combined of metals exposure on PE, with adjustments made for the maternal education and gestational age. Posterior inclusion probabilities (PIPs) of metals in the BKMR model are presented in Table 7. Notably, Cu and Cd have larger PIPs, indicating the greater their relative greater importance in influencing PE. Based on the results of PCA, Mn, Ni, Cu, Pb, As, and Hg were grouped together, while Cd and Zn formed another group. The group PIPs surpassed 0.5 among all groups, whereas Cd in group 1 and Hg in group 2 had larger conditional PIPs.

**Table 7:** Posterior inclusion probabilities of metals in BKMR model.

Metals	PIPs	Group	Group PIPs	Conditional PIPs
Zn	0.51	1	1	0.00
Mn	0.37	2	1	0.00
Ni	0.96	2	1	0.25
Cu	1.00	2	1	0.00
Pb	0.54	2	1	0.00
As	0.21	2	1	0.00
Cd	1.00	1	1	1.00
Hg	0.59	2	1	0.75

PIPs, posterior inclusion probabilities.

**Figure 2:**
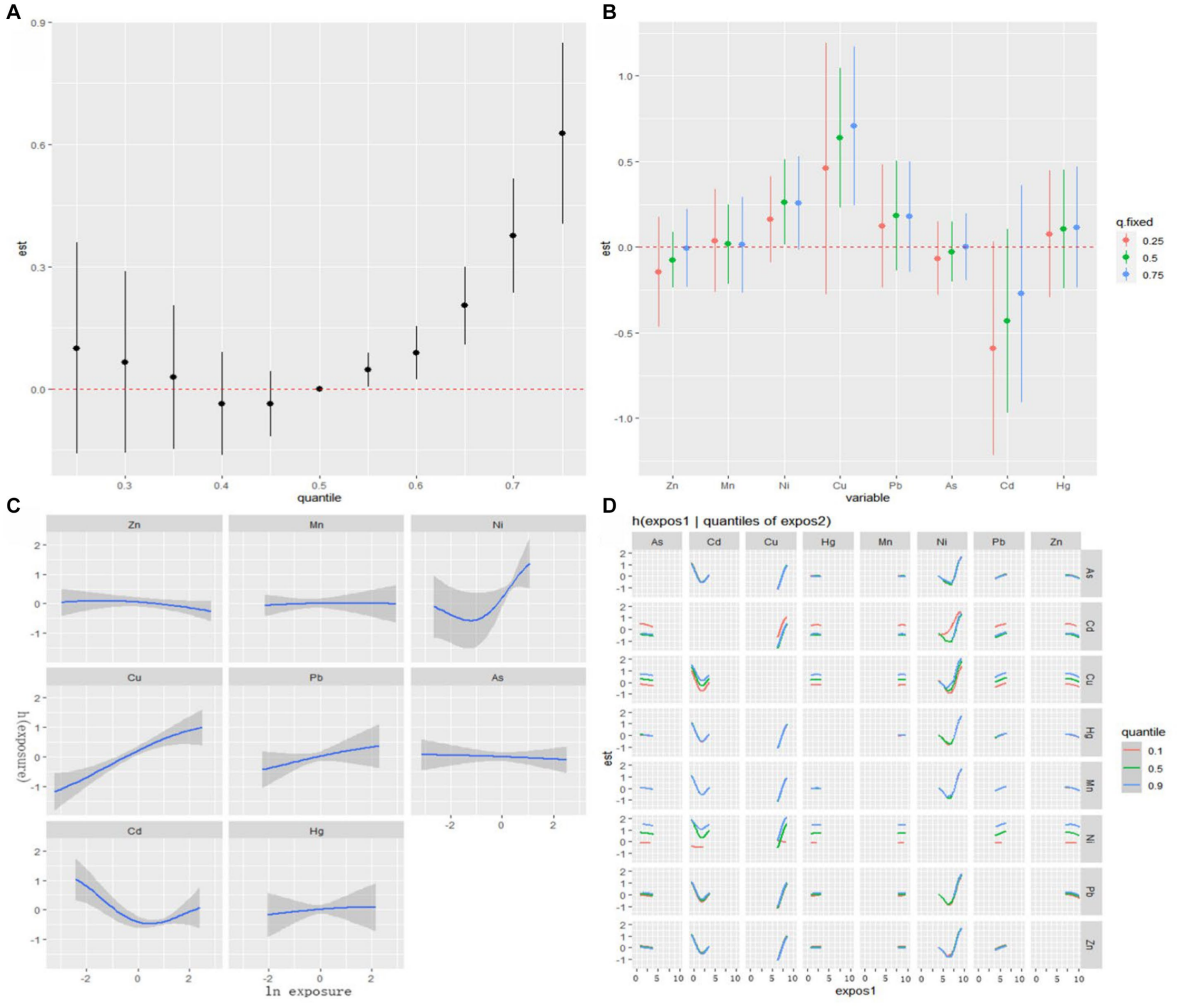
Associations between serum metals and PE among the study population by BKMR model. The model is adjusted for maternal education and gestational age. **(A)** The cumulative effect of the serum metals (estimates and 95% CIs). **(B)** The single-exposure effect (estimates and 95% CIs). **(C)** Univariate exposure-response functions and 95% confidence bands for each metal with the other metals fixed at the median. **(D)** Bivariate exposure-response functions.

The visualization of the BKMR model is depicted in Figure 2A. Cumulative toxic effect of metals is shown in Figure 2A, indicating a statistically significant overall effect when all metals were above their 50th percentile compared to when all metals were at their median values. Thus, elevated exposure could be associated with an increased risk of PE. The single effect of metals was explored by estimating the change in the association of a single metal with PE when it is positioned at the 25th and 75th percentiles, while the other metals were placed at the 25th, 50th, and 75th percentiles, respectively (Figure 2B). Notably, Ni and Cu exhibit a significant positive effect, with concentrations from the 25th to the 75th percentile associated with a significant increase in the risk of PE. To investigate the potential nonlinear relationship, exposure-response cross-sections for single metals were plotted, while fixing the levels of other metals at the median (Figure 2C). The plot suggested nonlinear effects of Ni and Cd, whereas, it showed a linear effect of Cu, while an increase in Cu levels is significantly associated with an increased risk of PE. To further investigate the potential relationship between metals, the bivariate exposure-response curve was plotted (Figure 2D). The curve illustrated the exposure-response relationship of one metal when the level of another metal fixed at the 10th, 50th and 90th percentiles, while remaining 6 metals were all fixed at the median. The plot indicates potential interaction between Cd and Ni as well as Cu and Ni. No evidence of interaction between other metals was observed based on parallel exposure-response relationships.

## 4 Discussion

In this study, we initially employed traditional unconditional logistic regression model to analyzed the relationship between multiple metals and PE. The associations between individual metals and PE showed that elevated serum levels of Mn, Cu, Pb, and Hg were associated with the risk and severity of PE, while elevated serum levels of Zn and As were associated with the risk of mild and severe PE, respectively. Our results are in-line with a number of previous studies which have reported the relationship between individual metals including Cu (26, 41), Pb (47), Hg (34), and As (33) and their association with PE. In contrary, there a study has reported an inverse dose–response relationship between Mn and PE (28), and a meta-analysis found significantly lower serum levels of Zn in PE patients (30). These discrepancies might be attributed to variations in the study population, region, presence of other metals and influencing factors, thus further analysis is needed. The logistic regression results indicated a dissimilar connection between Cd and mild or severe PE, so we hypothesized that there might be a nonlinear relationship between Cd and PE, which was explored in subsequent BKMR analyses.

Considering the current situation of metal pollution in Dongguan, we hypothesized that people are not simply exposed to a single metal, but to multiple metals at the same time. Therefore, we investigated the relationship between the overall effect of multiple metals and PE, and our findings revealed that an elevated overall score of metals was associated with the risk and severity of PE, indicating an association between exposure to multiple metals and PE. A study conducted in Taiyuan, China provided supporting evidence for similar results (35). The results from multivariate logistic regression showed that elevated concentrations of Cu and Pb were associated with the risk and severity of PE, while elevated concentration of Hg was associated with risk of severe PE. However, existing studies on the effect of Cu on PE have yielded inconsistent. Some studies align with our results, suggesting a relationship between Cu and PE (26, 41). Moreover, maternal blood pressure was positively correlated with the concentration of Cu (48). In contrast, a study in Bangladesh reported significantly reduced concentration of Cu in PE patients (49), and a study in Saudi Arabia proposed that the reduction of Cu might be one of the causes of PE (50). Regarding the association of Pb (51) and Hg (34) with PE, our results are consistent with some studies while conflicting with others that found null associations between Pb, Hg, and PE (36, 43). Notably, most available studies have utilized traditional statistical analyses, such as logistic regression and linear regression, the outcomes of which may be influenced by sample size and interactions among exposure factors.

The Spearman correlation analysis revealed highly significant correlations between serum concentrations of various metals. Subsequently, we utilized PCA for dimensionality reduction of metals and found that PC1 was predominated by Hg, Pb, Mn, Ni, Cu, and As, while, PC2 was predominated by Cd and Zn. Our study supported the association of PC1 with increased risk of PE, suggesting that pregnant women exposed to a combination of these metals might face an increased risk of developing PE. Correlations between combination of metals have also been reported in previous studies, with one study reporting that levels of Cu, Zn, Mg, and Mn were positively associated with Pb and Cd (52). It is known that toxic heavy metals such as Hg, Pb, and Cd might interfere and compete metabolically with essential metals such as Cu, Zn (53, 54), and Zn has been recognized for its protective role against Cd toxicity (55). Thus, when multiple metals are exposed simultaneously, there may be antagonistic, competitive, and promoting relationships between them, rather than a singular metal effecting the human body (56, 57). Pregnancy represents a unique physiological period, characterized by distinct sensitivity and adaptability compared to non-pregnant individuals, thus the effect of metals during this period may be more complicate. Establishment of inter-metal relationships may suggest a disturbance in element homeostasis among PE patients, potentially operating through shared pathways. More research is needed to explore the mechanism of interaction among metals in pregnant women and their role in PE.

In this study, we also employed a novel nonparametric BKMR model to further investigate the relationship between multiple metals and PE. The BKMR model unveiled a significant positive cumulative effect of serum metals on prevalence of PE when concentrations exceeded the 50th percentile. It is known that, if exposure surpasses a critical level, both essential and non-essential metals could exert a wide range of toxic effects on living systems (14, 58), including immune system dysfunction (59), reproductive performance (60), multifunction of neuronal systems (61), cancers (62), and induction of oxidative stress (63, 64), which may explain the cumulative effect of metals on the development of PE. The bivariate cross-sections of exposure-response functions indicated potential interactions between Cd and Ni, as well as Cu and Ni. Previous studies have suggested that certain metals, such as Cd, Cu and Ni can act as endocrine disruptors by mimicking the action of estrogens, thus metal ions with “estrogenic activity” are also termed as metalloestrogens (MEs) (65, 66). MEs may influence estrogen receptor function by binding to cellular estrogen receptors, thereby mimicking the action of physiological estrogens. This modulation of the hormonal status in the organism by MEs can induce disturbances in human organism homeostasis (67–69). The shared mechanism of action may explain the observed interactions between metals.

The strength of BKMR lies in its capability to address both the cumulative mixture effect and the dose–response impact of individual metal when other metals fixed a particular percentile (70). Our study identified a significant positive single effect of Cu and Ni on the risk of PE. It is suggested that the association between PE and serum Cu and Ni is stable and not affected by other metals. The findings for Cu in the BKMR model were consistent with the traditional logistic regression, both indicating that Cu exposure increases the risk of developing preeclampsia. A meta-analysis also supported the notion that plasma or serum Cu level in PE patients was significantly higher than that in healthy pregnant women (71). However, a systematic review indicated that Cu was associated with PE, but the levels of Cu leading to increased risk of PE varied across regions and economic development (72). Cu is an essential trace element that is involved in many biochemical processes and the function of several cuproenzymes and also acts as a powerful antioxidant to protect cells from damage. However, an excess of Cu can harm cells due to its potential to catalyze the generation of toxic reactive oxygen species (22). In conclusion, we propose the existence of a safe dose range of Cu in pregnant women, emphasizing that elevated levels of Cu are associated with the development of PE. The BKMR model indicated nonlinear effects of Ni and Cd, providing an explanation and correction of anomalous results in logistic regression. A study in South Africa showed no significant differences in the hair and serum levels of Ni between the PE group and the control group (42). Ni as a metalloestrogen, may impact on PE by modulating the hormonal status, however, further research is needed to explore to potential relationship. A study has found that elevated Cd levels in maternal circulation could potentially increase the risk of PE (32), whiles another study indicated that there were no significant differences in the plasma concentrations of Cd between different concentration groups (43). An *in vivo* study even reported Cd-induced immune abnormalities, possibly contributing to PE pathogenesis and offering insights into treatment strategies (73).

A significant strength of our study lies in its focus on exploring the association of multiple metals with PE, aligning more closely with the natural exposure of pregnant women to these metals. Moreover, we employed novel and flexible statistical methods (BKMR), allowing us to quantify and visualize the cumulative effect of the serum metals, investigation of dose–response relationship, and overcome the limitations associated with traditional analyses including challenges of high degree of correlation between compounds. While most of the current studies on metal toxicity predominantly focus on occupational groups, our research emphasizes the potential harm of metal exposure to the general population in heavily polluted areas. Our study was conducted in an electronics manufacturing city with varying degrees of metal pollution, local pregnant women may have a higher exposure to metals. Therefore, our study shed lights on the health effects of regional metal pollution on the pregnant women living in the area. Since our study is pre-exploratory and the sample size was relatively small, limiting our ability to assess subgroup effects. However, these preliminary findings provide valuable insights and encourage us to establish a cohort study in the later stage to study this issue further with expanded sample size. Moreover, subsequent research is needed to investigating the primary intake routes and sources of metals in pregnant women, contributing to the management of metal exposure and the prevention of PE.

## 5 Conclusion

This study unveiled a potential positive cumulative effect of serum metal levels on the risk of PE, with a particular emphasis on Cu as a potential risk factor for the onset and exacerbation of PE. Our findings suggest that pregnant women should maintain vigilance regarding the combined exposure to multiple metals, especially concerning elevated levels of Cu, Pb, Hg, and Ni. The results of this study offer valuable insights for directing future research on this issue and provide an additional foundation for preventing and treating PE. Nevertheless, larger cohort studies and experimental studies are required to investigate the risk of PE associated with exposure to multiple heavy metals. Furthermore, these studies should delve into potential interactions among different metals and elucidate the underlying mechanisms influencing the effects of metals on PE.

## Data availability statement

The original contributions presented in the study are included in the article/supplementary material, further inquiries can be directed to the corresponding author.

## Ethics statement

The studies involving humans were approved by the Ethics Committee of the Songshan Lake Central Hospital of Dongguan City and the Ethics Committee of the School of Public Health of Lanzhou University (audit no. IRB18010101). The studies were conducted in accordance with the local legislation and institutional requirements. The participants provided their written informed consent to participate in this study.

## Author contributions

JH: Conceptualization, Data curation, Investigation, Writing – original draft, Writing – review & editing. YP: Conceptualization, Investigation, Writing – review & editing. YDu: Data curation, Investigation, Writing – review & editing. HL: Investigation, Writing – review & editing. XW: Investigation, Writing – review & editing. SH: Investigation, Writing – review & editing. SA: Investigation, Writing – review & editing. YDa: Conceptualization, Funding acquisition, Writing – review & editing.
